# Neuronal viability/astrocyte activity ratio in the dorsolateral prefrontal cortex as a biomarker of Alzheimer’s dementia: A proton magnetic resonance spectroscopy study

**DOI:** 10.1002/alz.090573

**Published:** 2025-01-09

**Authors:** Shreya Jha, Edgardo Torres‐Carmona, Yusuke Iwata, Clement Ma, Ariel Graff‐Guerrero, Corinne E. Fischer, Benoit H. Mulsant, Bruce G. Pollock, Tarek K. Rajji, Sanjeev Kumar

**Affiliations:** ^1^ University of Toronto, Toronto, ON Canada; ^2^ Centre for Addiction and Mental Health, Toronto, ON Canada; ^3^ Campbell Family Mental Health Research Institute, Centre for Addiction and Mental Health, Toronto, ON Canada; ^4^ Temerty Faculty of Medicine, Department of Psychiatry, University of Toronto, Toronto, ON Canada; ^5^ Keenan Research Centre for Biomedical Science, Li Ka Shing Knowledge Institute, St. Michael's Hospital, Toronto, ON Canada; ^6^ Institute of Medical Sciences, University of Toronto, Toronto, ON Canada; ^7^ Department of Psychiatry, Temerty Faculty of Medicine, University of Toronto, Toronto, ON Canada

## Abstract

**Background:**

N‐acetyl‐aspartate (NAA) and myo‐inositol (mI) are neurometabolites reflecting neuronal viability and astrocyte activity, respectively. These can be quantified in vivo using proton magnetic resonance spectroscopy (1H‐MRS). Previous studies have suggested that these metabolites could serve as biomarkers for Alzheimer’s disease dementia (AD). NAA and mI levels were compared in the dorsolateral prefrontal cortex (DLPFC) between AD and cognitively healthy control participants to assess for significant differences, assess if NAA/mI ratio can distinguish the two groups, and to explore the relationship between metabolites and cognition.

**Method:**

Participants with AD and HCs over 55 years old were included. Bilateral NAA and mI levels were quantified in the DLPFC using 3T proton magnetic resonance spectroscopy (point‐resolved spectroscopy, echo time=35ms). NAA and mI levels were estimated using LCmodel version 6.3 and reported in H_2_O ratios i.e. NAA/ H_2_O or mI/H_2_O.

**Result:**

The study included 64 participants, 41 with AD (mean (SD) age = 75.2 (7.2) years, females = 58.5%), and 23 HC (age = 72.2 (7.7) years, females = 60.1%). Bilateral NAA levels in the DLPFC were lower in AD vs. HC, (t(62)=‐2.5, p =0.02); however, mI levels in the DLPFC did not differ between the two groups t(62)=1.0, p=0.35. NAA/mI ratio was lower in AD vs. healthy (t(62)= ‐2.368, p =0.02). The NAA/mI ratio at a cut off value of 1.69 showed 59% sensitivity and 87% specificity at distinguishing AD from HC. NAA levels were associated positively with global cognition.

**Conclusion:**

DLPFC NAA is decreased in AD and is associated with cognition. NAA/mI ratio shows good specificity in distinguishing AD from the control group, suggesting its role in complementing other biomarkers in ascertaining AD. Future studies should prospectively evaluate the clinical utility of NAA/mI ratio as a biomarker in AD.

